# Supplementary material for: The Construction and Exploration of a Comprehensive MicroRNA Centered Regulatory Network in Foxtail Millet (Setaria italica L.)
Source: Front Plant Sci. 2022 May 6;13:848474. doi: 10.3389/fpls.2022.848474 (PMC9121102; doi:10.3389/fpls.2022.848474)
Supplement: Supplementary file 4 [file Data_Sheet_4.zip › Sit-miR408_Seita.1G092300.1_281_TPlot.pdf]

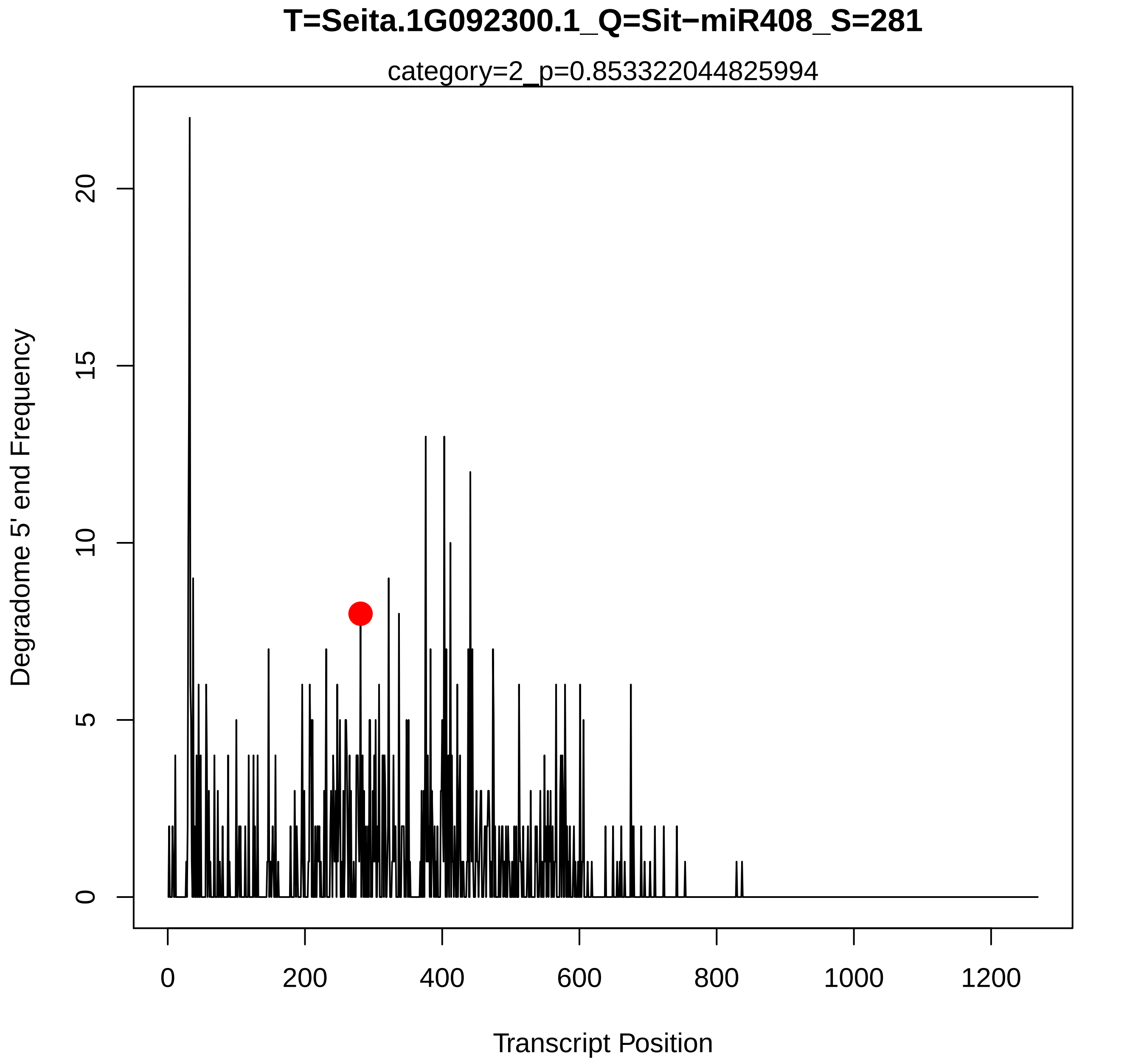

T=Seita.1G092300.1_Q=Sit−miR408_S=281
category=2_p=0.853322044825994
Degradome 5' end Frequency
Transcript Position
0
5
10
15
20
0
200
400
600
800
1000
1200
